# Interventions to Ameliorate the Psychosocial Effects of the COVID-19 Pandemic on Children—A Systematic Review

**DOI:** 10.3390/ijerph18052361

**Published:** 2021-02-28

**Authors:** Katharina Boldt, Michaela Coenen, Ani Movsisyan, Stephan Voss, Eva Rehfuess, Angela M. Kunzler, Klaus Lieb, Caroline Jung-Sievers

**Affiliations:** 1 Institute for Medical Information Processing, Biometry and Epidemiology—IBE, Chair of Public Health and Health Services Research, LMU Munich, Elisabeth-Winterhalter-Weg 6, 81377 Munich, Germany; kboldt@ibe.med.uni-muenchen.de (K.B.); coenen@ibe.med.uni-muenchen.de (M.C.); ani.movsisyan@ibe.med.uni-muenchen.de (A.M.); svoss@ibe.med.uni-muenchen.de (S.V.); rehfuess@ibe.med.uni-muenchen.de (E.R.); 2 Pettenkofer School of Public Health, 81377 Munich, Germany; 3 Department of Psychiatry and Psychotherapy, University Medical Center, 55131 Mainz, Germany; Angela.Kunzler@lir-mainz.de (A.M.K.); klaus.lieb@unimedizin-mainz.de (K.L.); 4 Leibniz Institute for Resilience Research (LIR), 55122 Mainz, Germany

**Keywords:** COVID-19, pandemic, child, mental health, intervention

## Abstract

The aim of this study was to identify interventions targeting children and their caregivers to reduce psychosocial problems in the course of the COVID-19 pandemic and comparable outbreaks. The review was performed using systematic literature searches in MEDLINE, Embase, PsycINFO and COVID-19-specific databases, including the CDC COVID-19 Research Database, the World Health Organisation (WHO) Global Database on COVID-19 Research and the Cochrane COVID-19 Study Register, ClinicalTrials.gov, the EU Clinical Trials Register and the German Clinical Trials Register (DRKS) up to 25th September 2020. The search yielded 6657 unique citations. After title/abstract and full text screening, 11 study protocols reporting on trials planned in China, the US, Canada, the UK, and Hungary during the COVID-19 pandemic were included. Four interventions targeted children ≥10 years directly, seven system-based interventions targeted the parents and caregivers of younger children and adolescents. Outcome measures encompassed mainly anxiety and depressive symptoms, different dimensions of stress or psychosocial well-being, and quality of supportive relationships. In conclusion, this systematic review revealed a paucity of studies on psychosocial interventions for children during the COVID-19 pandemic. Further research should be encouraged in light of the expected demand for child mental health management.

## 1. Introduction

Crises such as disease outbreaks are usually accompanied with stress and uncertainty throughout the whole population. However, depending on the character of a disease outbreak, particular challenges present in specific vulnerable groups. In the context of the SARS-CoV-2 outbreak, which has been officially declared a pandemic by the World Health Organization (WHO) as of 11 March 2020, children and families are particularly affected by negative consequences. These consequences encompass containment measures, such as childcare and school closures with separation and isolation from children’s social groups and friends, contact restrictions, quarantines, the loss of freedom and opportunities for movement and play, increased boredom, the discrimination against affected families, insecurity of adult caregivers with increased familiar stress and domestic conflicts, adverse effects on children’s educational opportunities and impacts on parent’s employment [1,2,3].

Hence, increased anxiety and depression rates are common responses among children and adolescents in affected countries [2,4,5,6]. Depending on the study and setting, the proportion of children and adolescents reporting mental health issues above risk thresholds of distress, particularly anxiety and depression, can range up to 60% [7,8,9]. Children with pre-existing mental health conditions and of more deprived populations seem to be particularly vulnerable in this regard. At the same time, psychiatric attendances seem to be reduced in the pandemic which can be interpreted as unmet health care needs.

Since children are at a particularly vulnerable stage of life, special attention should be paid to psychosocial interventions for children and adolescents in crisis situations with regard to mental illness prevention and mental health promotion. Possible interventions may target, for example, the ability to build resilience, express and regulate emotions, pursue meaningful and relaxing activities, and thus develop alternatives to risk behaviors [10].

These interventions may help combat mental illness in the future and prevent the enormous personal and societal burdens associated with it. In this context, early engagement seems to be crucial to prevent long-term consequences such as substance abuse, lower educational achievement, and development of violence [11,12].

As in other crises, WHO and other organizations emphasize the importance of multisectoral, early psychosocial support for the young and provide guiding information materials on the topic [13,14,15].

However, to date, evidence on the existence, specific approaches, content, and possible effects of psychosocial interventions to prevent or manage mental health outcomes of children in the course of the COVID-19 pandemic is sparse. The objective of this review was therefore to identify interventions that target children, their caregivers and/or families, in order to reduce the psychosocial impacts caused by the COVID-19 and comparable disease outbreaks, as well as associated control measures (e.g., quarantine).

## 2. Materials and Methods

This systematic review was performed based on a protocol pre-registered on the Open Science Framework (OSF): https://osf.io/chpvk.

### 2.1. Search Methods for the Identification of Studies

We performed searches on 25 September 2020 in MEDLINE, Embase, and PsycINFO, using a combination of terms related to disease outbreaks and their spread, the population of interest, psychosocial outcomes and interventions (see Appendix A, Appendix B, Appendix C, Appendix D, Appendix E, Appendix F, Appendix G, Appendix H and Appendix I). We additionally searched COVID-19-specific databases, including the Centers for Disease Control and Prevention (CDC) COVID-19 Research Articles Downloadable Database, the WHO Global Database on COVID-19 Research and the Cochrane COVID-19 Study Register, as well as clinical trials registries, namely ClinicalTrials.gov, the EU Clinical Trials Register and the German Clinical Trials Register (DRKS).

Our search strategy was reviewed by an experienced information specialist. Database searches were limited to studies published in English, German, Russian, and French. No restrictions based on the year of publication and publication format were applied.

Inclusion and exclusion criteria for studies are described in Table 1.

### 2.2. Study Selection

After removal of duplicate studies, we performed a multistage screening process to select those studies which met the inclusion criteria:

- Stage 1, screening of titles and abstracts: One review author screened the titles and abstracts of all identified records (KB). Twenty percent of all titles and abstracts were independently assessed by a second review author (CJS, AM, MC, SV) and in case of disagreement discussed with a third reviewer (CJS, AM, MC, SV). At this stage, an inclusive approach was adopted and all unclear studies were taken forward to full text screening.
- Stage 2, screening of full texts: Two review authors (KB, CJS, AM, MC, SV) independently screened the full texts of all studies selected by at least one review author at stage 1. Uncertainties were resolved through discussion or by consulting a third review author (KB, CJS, AM, MC, SV).

EndNote was used to collect and de-duplicate the studies. For the screening of titles and abstracts we used Rayyan web-based application for facilitating citation screening for systematic reviews [16]. At stage 2 of the screening process, we documented the reasons for exclusion using Microsoft Excel spreadsheets.

### 2.3. Data Extraction, Management, and Synthesis

One review author (KB) extracted study data and characteristics to an Excel-based data extraction form (see Appendix J). A second review author (CJS) checked for completeness and correctness. We synthesized the findings of the included studies narratively and with tables.

## 3. Results

### 3.1. Results of the Search

**Figure 1:**
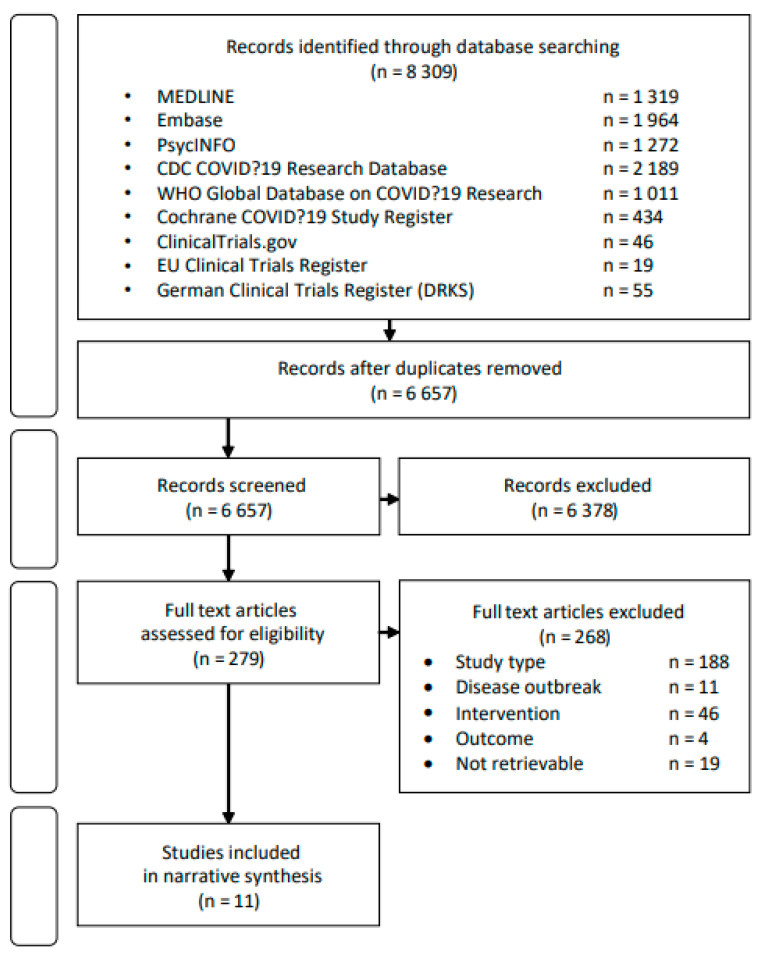
Preferred Reporting Items for Systematic Reviews and Meta-Analyses (PRISMA) flow chart.

Overall, our systematic searches yielded 6657 unique records. After title and abstract screening, 279 records were taken forward for full text screening (Figure 1). Of these, 260 full text papers were assessed for eligibility. Most of these were non-empirical studies such as commentaries, editorials, reviews, news and adapted recommendations that emphasize the importance of preparing for a mental health crisis in the context and aftermath of the COVID-19 pandemic. The full texts of 19 papers could not be retrieved.

### 3.2. Included Studies

After title/abstract and full text screening, 11 papers met our eligibility criteria. These papers are summarized in Table 2 and Table 3. All manuscripts report on study protocols. Four protocols were registered in China, three in the US and two in Canada, and one in the UK and one in Hungary. All studies are planned in the COVID-19 setting and none identified in previous SARS, MERS, or H1N1 influenza outbreaks. Four study interventions addressed children directly; in two and five studies the interventions were targeted at children plus parents/caregivers combined or at parents/caregivers only. Two interventions are intended for face-to-face delivery, nine studies are designed as digital interventions.

Four of the included papers were study protocols for randomized controlled trials (RCTs) on digital interventions for children aged ≥10 years that aimed to reduce symptoms of anxiety or to improve the emotional well-being or the ability to support others during the COVID-19 outbreak.

The study protocol by Chen [17] aims at assessing the effectiveness of an online Solution Focused Brief Therapy (SFBT) for adolescents. SFBT is a short-term psychotherapy option that builds on positive emotion-evoking, resource- and future-oriented principles of behavior modification [18]. The second included protocol by Zheng [19] plans to test a live-streaming application for children that offers a Recess and Exercise Advocate Program (REAP) during online learning classes in the time of school closure and home confinement. REAP is a digital platform that allows students to watch stay-at-home workouts, record short videos, or take photos related to their own physical exercise and upload these to the app, using smartphones. Tymofiyeva [20] aims at evaluating the efficacy of Training for Awareness Resilience and Action (TARA), a neuroscience-based program for adolescent anxiety and depression informed by mindfulness-based therapy, yoga and modern psychotherapeutic techniques [21]. Pavarini [22] plans to co-produce and deliver an online peer support training together with young people and the peer support charity YouthEra. Although some of these studies, once completed, may include some adolescents aged 18 years and above (thus outside of our inclusion criteria), the majority of study participants will likely be within our age range of 0–17 years as defined by the inclusion criteria.

We found seven papers that describe system-based interventions targeted at parents, caregivers, and families. Two trial protocols present behavioral interventions with a special focus on pregnant women and mothers of infants, addressing perinatal anxiety and/or depression during the COVID-19 pandemic.

The planned study by Green [23] aims at assessing the effectiveness of an augmented version of a Cognitive Behavioral Group Therapy (CBGT). Enrolled women will undergo treatment that is based on cognitive and behavioral strategies for treating perinatal/postpartum anxiety and additionally addresses critical COVID-19-related worries and impacts. Huang [24] plans to evaluate the effect of an internet-based Cognitive Behavior intervention (iCBT) which comprises mental health education, cognitive restructuring, problem-solving, behavior reinforcement, and relapse prevention strategies and techniques, tailored to a perinatal population. Participants will receive weekly text- or video-based self-help resources via a perinatal mental healthcare app. After studying each course material, the women will be asked to give digital feedback to the therapist.

Three further clinical trial protocols plan to apply cognitive and behavioral strategies to positively impact COVID-19-specific family distress, anxiety, depression or the parent–child and couple relationship: the planned trial by Monga [25] aims at assessing an adapted Virtual-Care Cognitive Behavioral Therapy (VC-CBT) delivered by a therapist. Parents will accompany their children into the first and last therapy sessions and join at the end of each session for a review of newly acquired skills and completed exposure tasks. The purpose of another research study by Ehrenreich-May [26] is to evaluate the group cognitive behavioral therapy named Unified Protocol for COVID-19 Parenting Stress (UP-COVID). Lin [27] plans to provide online psychological interventions for parents. Participants in a parent–child relationship intervention group will receive cognitive and behavioral group therapy sessions including mindfulness and emotion regulation training to improve parent–child communication. Parents assigned to a couple relationship intervention group will participate in group therapy sessions that will focus on problem-solving and marital relationship improvement.

The last two study protocols focus on digital interventions aiming at reducing caregivers’ level of stress and improving the social, emotional and behavioral well-being of parents and children during the pandemic: Miklósi [28] plans to examine the effect of an internet-based parenting training and to determine the optimal program structure. The program will provide short psychoeducation videos and written materials focusing on parents’ and children’s stress. Worksheets, quizzes, and feedback forms will be involved to increase the participants’ engagement with the training program. Parents will have flexible access to the online platform. In a protocol for a pilot clinical trial, Francis [29] describes a tele-wellness supported digital toolkit through which parents and other caregivers will receive daily parenting support and self-care resources. Parents will additionally be asked to provide their child access to digital learning games in the app.

## 4. Discussion

With this systematic review, we identified interventions for children, their families and/or caregivers that may mitigate psychosocial effects caused by the COVID-19 pandemic or similar epidemics in children. No completed studies on the subject could be identified. However, we included 11 study protocols that aim at assessing interventions developed in response to the ongoing COVID-19 pandemic. The included child-based interventions mainly address anxiety and emotional problems in the targeted individuals, whereas the system-oriented approaches assess plan direct and indirect outcomes, such as stress, depression or self-efficacy on one hand, and family conflict, communication, and relationships and child well-being on the other hand. The studies were planned and will be conducted in China, the US, Canada, UK, and Hungary. Why there were no previous studies on SARS, MERS, or H1N1 influenza outbreaks might be explained by a lack of awareness of and attention paid to the adverse impacts on children, possibly due to the relatively smaller extent, shorter duration, and lower intensity of enforced measures to contain the disease spread during these past outbreaks.

### 4.1. Population

The included protocols for planned intervention studies not only targeted children directly, but also parents and caregivers of children. All parent-oriented interventions use a system-based approach by which children’s psychosocial well-being is to be positively influenced by enhancing the caregivers’ emotional stability and parenting competence. Since rising levels of stress associated with emotional disturbance and irritability in parents are related to reduced closeness of family relationships and higher incidence of harsh or punitive parenting [30]. Insecure parent–child attachments, in turn, are associated with higher risk of children developing internalizing and externalizing behavior problems that may persist into later life [31,32]. A high-risk group are pregnant women, particularly vulnerable to develop anxiety and depressive symptoms that can cause psychological distress and adversely affect the early healthy growth and development of their children [33]. Parenting practices, coping mechanisms and family environment have been shown to affect children’s mental health throughout and well after disasters [34]. Parents therefore represent an important target group for intervention approaches during and after emergency situations, not only for the sake of their own but also their entire family’s psychological well-being.

### 4.2. Intervention Types

The most widely recognized psychotherapeutic technique in the context of reducing anxiety and affective disorders is Cognitive Behavioral Therapy (CBT). CBT, as applied in several included trial protocols (23–26), has shown to be effective in individual, group, and family formats. Although mostly delivered face to face, it can also be carried out via virtual teleconferences [35]. The efficacy of the latter in the reduction of symptoms of anxiety and depression in pediatric populations was recently reviewed, showing a medium effect size of internet-based CBT compared to waitlist control groups [36]. A web-based self-help approach of trauma-focused CBT, designed specifically to help children and parents recover from traumatic life events and improve disaster-related PTSD symptoms, performed well in another efficacy study [37]. Evidence-based, scalable virtual-care CBT programs targeted at youth and/or parents might therefore be of significant value during pandemic crises as well as during post-pandemic recovery periods.

In contrast to diagnostic, step-by-step therapeutic approaches, the SFBT treatment, as tested by Chen [17], encourages flexibility when incorporating underlying techniques in order to meet each client’s individual needs and achieve sustainable changes within a few therapy sessions. Empirical evidence shows its effectiveness in addressing internal psychological problems, such as anxiety and/or depression, across diverse populations and various settings, such as schools [38]. Due to its high level of adaptability, it might be a promising therapeutic approach to overcome mental health disorders in quickly evolving and unpredictable pandemic situations.

TARA, as proposed by Tymofiyeva [20], builds on elements of time-sensitivity and the creation of a platform for attention and behavioral motivation [21]. The mindfulness intervention has demonstrated maintained efficacy in reducing anxiety and depressive symptoms in adolescents [39]. As other types of therapy showed comparable symptom and behavior outcomes if delivered remotely, TARA might be another promising therapeutic approach to apply during pandemic-related quarantine measures.

Some study protocols [27,28,29] consider prevention-based psychological approaches to reduce family stress and enhance emotional well-being as well as relationship and parenting skills. Self-directed (SD) parenting interventions with varying levels of support through a therapist provide caregivers with support and self-care materials to acquire positive behavioral strategies. In a recent empirical research, parental psychological flexibility showed to be robustly linked to family functioning in the midst of a pandemic. Accordingly, parental inflexibility was predictive of greater COVID-19-related stress, leading to worsened family cohesion, higher family discord and reduced child and parent well-being [40]. Findings from another study emphasize the significant role of parenting stress in constructive parenting behavior and parent–child relationships during COVID-19 [30]. SD interventions have delivered supportive outcomes of decreased negative child behaviors, improved parenting behaviors and family interactions along with reduced parental stress [41]. Telehealth formats of the SD intervention Parent–Child Interaction Therapy (PCIT) [42] and Triple P [43] were found to be comparably effective. Online parent support programs therefore present potentially valuable public health approaches for helping families to overcome challenges of a large-scale crisis such as the COVID-19 pandemic.

The intervention approach proposed by Zheng [19] is physical activity-based. Physical activity (PA) is an adaptive coping strategy that can help to reduce anxiety levels and depressive symptoms [44,45]. For children, PA is closely linked to school hours and related activities such as active travel to school or organized sports [46]. As a consequence of school closures and stay-at-home orders in the wake of the COVID-19 pandemic, opportunities for children to meet movement behavior guidelines are compromised. First studies on the topic report on substantially decreased PA levels and increased screen time in youth since the initial outbreak [47,48,49]. Interventions such as the REAP may be promising approaches to encourage exercise uptake at home in order to counteract the lack of PA related to public health restrictions on the one hand, while reducing psychological symptoms, such as stress and anxiety, and improving well-being on the other hand.

Social and community support are generally discussed as critical protective factors for resilience and mental health in children [50]. Involuntary social isolation as a consequence of pandemic control measures is likely to result in higher levels of loneliness which in turn are associated with the onset of mental disorders [51]. Online peer support as proposed by Pavarini [22] can help young people to connect, provide and seek emotional and informational support as well as to share experiences and thereby reduce negative impacts of otherwise disrupted social networks. The effectiveness of online peer-to-peer interaction as a stand-alone intervention in improving mental health in youth has yet to be evaluated [52].

### 4.3. Intervention Delivery

The majority of the planned interventions will be provided over telecommunication platforms accessible via the Internet, such as Zoom. Some approaches will make use of digital mobile health (mHealth) applications. During pandemics with measures to contain transmission, digital solutions present promising ways with increasing impact to bridge the social distance and deliver health services to populations with eHealth literacy, i.e., the young, that may at the same time be vulnerable and underserved. An evolving evidence base supports the feasibility, acceptability and efficacy of digital mental health interventions in children and adolescents in non-crisis times [53,54,55,56]. The opportunities in eHealth or mHealth interventions lie in a simplified and low-threshold access, scalability and economic benefits [57]. However, their implementation in times of pandemic restrictions can pose challenges. Perrin and colleagues [58] outline situations where chaotic home environments and limited privacy during COVID-19 self-isolation and quarantine measures complicated telepsychology sessions. Other challenges discussed in the literature were related to the technology itself, as use and success of virtual prevention and treatment require suitable electronic devices and reliable home internet connection. Some authors expressed concerns regarding disparities in access to digital resources due to, for example, rural settings or socioeconomic status, urging for universal, equal and secure provision of telehealth services in order to unfold their potential to contribute to sustainable health equity in the long term [59,60]. Overall, the pandemic is a potential driver for digitalization in healthcare delivery.

### 4.4. Funding

Universities or university associated foundations or hospitals were named as sponsors for most of the studies. The study by Green et al. [23] was sponsored by a health care foundation named St. Joseph’s Healthcare Hamilton. For only one study by Pavarini et al. [22], it was explicitly mentioned that it was funded by an Urgent Response Fund, the “Economic, Social, Cultural and Environmental Impacts of COVID-19: Urgent Response Fund” by the Oxford University/Torch, UK. This study additionally drew on the expertise and resources of YouthEra, a non-profit organization with experience in youth empowerment and peer support training programs, and the McPin Foundation with the aim to transform mental health research. Thus, after 9 months from the onset of the pandemic, only one study was funded based on an explicitly mentioned funding structure for COVID-19 projects. On the other hand, no studies were funded by ministries or government research organizations which can be interpreted as a gap of early response funding resources in the field of childhood mental health projects in the context of pandemics.

### 4.5. Gaps

All study protocols present interventions that aim at behavior modification. There was little material on interventions that operate on a situational level. Roca and colleagues [61] present virtual evidence-based actions applied in schools in Spain during enforced closure periods serving as open doors to a safe environment for interactions with the aim of preventing child abuse. The strategy included, for example, dimensions of online dialogic workspaces and dialogic gatherings with students. The action plans were derived from school-based abuse prevention programs that have previously shown to be effective in providing knowledge and skills for children to understand concepts of abuse and in reducing the risk of violence. The authors point out on further potential actions inspired by existing school-based interventions. The evaluation of such as well as other structural prevention or promotion approaches in the current pandemic context is required in order to assess their efficacy throughout large-scale crises.

We found a lack of approaches that specifically address maltreatment and abuse of children. High levels of familial stress can result in impulsive or even aggressive behavior responses [62]. Physical isolation is likely to exacerbate already strained situations [46]. Several countries have reported on increased violence against women and children associated with the implementation of COVID-19 emergency measures [63]. In a recent perspective paper, Emezue [64] reviews evolving digital solutions at both micro- and macrolevel to mitigate domestic violence in the context of the current pandemic. The research highlights some web- and mobile-based interventions that have been proven effective in preventing violence and increasing safety for several survivor cohorts in pre-pandemic times and could also be adopted to the current crisis situation. In view of the significant violence risk exposure of children during home confinement, interventions against domestic violence need to be adapted and scaled up.

### 4.6. Strengths and Limitations

It is noteworthy that although we performed a very extensive review covering established health and psychological databases, COVID-19 databases as well as trial registries and yielded 6657 records, we were only able to include 11 study protocols and no completed study. The fact that reviews, even performed to a high scientific standard, result in no or few completed studies included is well described and acknowledged by organizations such as the Joanna Briggs Institute or Cochrane [65,66]. This may happen in areas of limited evidence, new topics, or very specific questions asked. To tackle this challenge in a pandemic, we set the frame very broad with a wide definition of the population, interventions, outcomes, study designs, and related pandemic settings. However, the fact that we only found 11 study protocols under these circumstances is a result per se. From our point of view, it reveals a knowledge gap in a field that will be of high relevance in the near future and pandemic aftermath. In addition, although we cannot base our strategies on effects of interventions, the benefit of this review is to identify, discuss and highlight the range of potentially promising strategies that could be used and upscaled in the future and to frame and set the research agenda in the field.

Some further limitations have to be put into account. Due to the fast-paced science related to COVID-19, there is a high publication frequency that makes this work a snapshot of the current but rapidly changing knowledge base. Moreover, a snapshot of what type of interventions researchers planned in the first semester of the pandemic which might not be the same strategy as in later stages of the pandemic. This early phase was also the reason some (*n* = 19) full texts could not yet be retrieved at the time of data extraction. Although the fact that all manuscripts report only study protocols, but not on completed studies, leaves the question unanswered whether the described approaches will also be effective or not.

Especially in the grey literature, we might have additionally missed interesting interventions and approaches not described in scientific publications, such as digital solutions in the format of mobile applications and others.

## 5. Conclusions

Since we expect a high burden of mental health impacts during and in the aftermath of the COVID-19 pandemic among the young, there is likely to be a high demand for pragmatic mental health management concepts and interventions. To address this need, different approaches would be helpful: on the one hand, we may draw on existing evidence generated during non-pandemic situations and adapt materials and approaches, such as information resources and mHealth tools, to the COVID-19 pandemic and future large-scale crises contexts. On the other hand, we have very specific issues to address such as home confinement settings, disruption of education and opportunities for socialization, absence of structured days for a long duration, boredom and lack of physical, extracurricular, and outdoors activities. Because of these reasons, we would need specific and well-developed interventions targeting psychosocial effects on children in the context of the COVID-19 pandemic. At the same time, intervention programs that increase exercise, education, and socialization could include psychosocial outcomes as secondary objectives to enrich the knowledge in the field. Early financial support programs should integrate mental health prevention as part of the pandemic response. Furthermore, the effectiveness of these interventions should be evaluated carefully, to allow for evidence-based decisions in future pandemics on how to mitigate the impact of these crises on the mental health status of children.

## Figures and Tables

**Table 1:** Inclusion and exclusion criteria.

	Inclusion Criteria	Exclusion Criteria
Population	Children aged 0–17 years and/or their families/primary caregivers - Living in low-, middle- or high-income countries with COVID-19/SARS/MERS or H1N1 influenza pandemic or outbreak	Children and/or their families/primary caregivers - In pandemic situations with haemorrhagic fevers (including Dengue and Ebola) - Admitted to hospital wards - With other acute viral and bacterial respiratory infections
Intervention/exposure	Interventions aimed at ameliorating the psychosocial effects of pandemic situationsIntervention types, e.g.,: - Training courses - Exercises - Teaching materials and information - Support groups Intervention delivery modes, e.g.,: - Face to face, online, or smartphone-based - Individual, group, population-based - Child-based, system-basted	Interventions aimed at - Behavioral modification of disease spread (containment, protection, mitigation) - Inpatient settings - Patients with specific diseases, e.g., chronic diseases
Comparison	Treatment as usual (TAU) - Alternative treatment - Waitlist control - No treatment	/
Outcomes	Primary health outcomes, i.e.,: - Self-efficacy - Behavioral problems - Mental health problems - Stress - Maltreatment Secondary implementation outcomes, e.g.,: - Attendance - Engagement	Somatic outcomes of SARS-CoV-1, SARS-CoV-2, MERS-CoV-1, and H1N1 influenza infectionAny other outcomes not listed above, e.g.,: - Physical activity - Concentration - School grades
Study designs	Any study or protocol using an empirical study design, including: - Randomized controlled trials - Cohort studies - Cross-sectional studies - Qualitative studies - Mixed-methods studies - Case series with ≥10 persons - Case series with <10 persons (only for COVID-19, SARS, and MERS)	Non-empirical studies, including: - Commentaries - Letters - Editorials - Overviews Mechanistic dataAnimal studiesLaboratory studies

**Table 2:** Characteristics of included studies.

First Author(s)	Journal/Database	Date Published	Planned Study Completion Date	Study Type According to Protocol	Population and Context	Country
Chen S [17]	Trials	13 May 2020	NR	Study protocol for a randomized delayed crossover open-label controlled trial	Target group: Children (11–18 years) Planned number: 76 Condition/context: Manifesting anxiety symptoms, Generalised Anxiety Disorder-7 score ≥10	China, Beijing
Zheng Y [19]	ClinicalTrials.gov	16 March 2020	29 March 2020	Study protocol for a cluster-randomized controlled trial	Target group: Children (12–13 years) Planned number: 954 Condition/context: Under home confinement, enrolled in online learning courses	China
Tymofiyeva O [20]	ClinicalTrials.gov	14 September 2020	20 May 2020	Study protocol for a randomized group treatment, open-label, waitlist-controlled clinical trial	Target group: Children (14–18 years) Planned number: 21 Condition/context: NR	US, California
Pavarini G [22]	ISRCTN registry	29 May 2020	10 July 2020	Study protocol for a pilot randomized controlled trial	Target group: Children (16–18 years) Planned number: 100 Condition/context: NR	UK
Green S [23]	ClinicalTrials.gov	3 August 2020	1 August 2022	Study protocol for an open-label clinical trial	Target group: Women (18–45 years) Planned number: 120 Condition/context: Pregnant or up to six months postpartum, primary diagnosis of an anxiety disorder	Canada, Ontario
Huang HF [24]	International Clinical Trials Registry Platform (ICTRP)	31 May 2020	1 April 2022	Study protocol for a multi-center randomized controlled trial	Target group: Women (18 + years) Planned number: 300 Condition/context: Pregnant, Edinburgh Postnatal Depression Scale score >9 during the third trimester	China, Shanghai
Monga S [25]	ClinicalTrials.gov	29 May 2020	1 June 2021	Study protocol for an open-label clinical trial	Target group: Children (12–17 years) Planned number: 20 Condition/context: Primary diagnosis of an anxiety disorder	Canada, Ontario
Ehrenreich-May J [26]	ClinicalTrials.gov	16 June 2020	7 November 2020	Study protocol for a randomized open-label clinical trial	Target group: Parents (18+ years) Planned number: 68 Condition/context: Mild or greater elevation on screening measures of anxiety, depression and/or traumatic stress; parent of a child aged 6–13	US, Florida
Lin X [27]	ClinicalTrials.gov	9 July 2020	31 January 2022	Study protocol for a randomized clinical trial	Target group: Parents Planned number: 300 Condition/context: Parent of a student in primary and secondary school	China, Beijing
Miklósi M [28]	ISRCTN registry	23 May 2020	15 December 2020	Study protocol for a randomized controlled trial	Target group: Parents Planned number: 300 Condition/context: Parental stress; parent of a child aged <18	Hungary
Francis L [29]	ClinicalTrials.gov	1 July 2020	September 2021	Study protocol for a pilot clinical trial	Target group: Parents, other caregivers Planned number: 270 Condition/context: Family Child Care Home (FCCH) providers, parent of child aged 3–6 served by enrolled FCCH provider	US, Maryland

**Table 3:** Characteristics of interventions.

First Author(s)	Description of Specific Measure(s)	Format	Mode of Delivery	Comparison Group	Primary Outcome Measures	Follow-Up (Baseline)
Chen S [17]	Solution Focused Brief Therapy (SFBT)	Sessions: 2–4 Duration: 2 weeks	Online platforms, such as Zoom	2–4 sessions of counselling service	Anxiety (Generalised Anxiety Disorder-7, State-Trait Anxiety-Inventory)	2 weeks, 1 month
Zheng Y [19]	Recess and Exercise Advocate Program (REAP, llive-streaming stay-at-home workouts)	Sessions: NR Duration: NR	Mobile application	Online health information + stay-at-home workout videos	Anxiety (Spence Children’s Anxiety Scale)	2 weeks
Tymofiyeva O [20]	Training for Awareness, Resilience, and Action (TARA, meditation training)	Sessions: NR Duration: 12 weeks	Partially over the online platform Zoom in a group of 12 adolescents	Waitlist control group (9 adolescents)	Emotional problems (Strengths and Difficulties Questionnaire Items)	12 weeks
Pavarini G [22]	Peer support training	Sessions: 5 (à 4 h) Duration: 5 days	Online	Waitlist control group	Motivation to provide peer support, perceived support giving skills, frequency of support provided (Adolescent Social Connection and Coping During COVID Scale Items) Compassion towards others (Compassionate Engagement and Action Scale-Compassion for Others Scale) Connectedness to peers (Inclusion of Other in the Self Scale)	1 week (all) 2, 3, 4 weeks (intervention arm)
Green S [23]	Cognitive Behavioral Group Therapy (CBGT)	Sessions: 8 (à 2 h) Duration 8 weeks	Groups of 6 people	/	Anxiety (State-Trait Inventory of Cognitive and Somatic Anxiety)	8 weeks, 3 months
Huang HF [24]	Internet-based Cognitive Behavior Therapy (iCBT) (text- or video-based self-help resources)	Sessions: 7 Duration: 6 weeks	Mobile application	Routine treatment	Perinatal depression (Edinburgh Postnatal Depression Scale)	3. trimester + 6 weeks, 3, 6, 9 months, 1 year postpartum
Monga S [25]	Virtual-Care Cognitive Behavioral Therapy (VC-CBT)	Sessions: 12 (à 1 h) Duration: 12–16 weeks	Online platform Zoom	/	Family impact (CoRonavIruS Health Impact Survey) Efficacy of Virtual-Care CBT Intervention (Child and parent-report Screen for Child Anxiety Related Disorders) Clinician virtual-care experience (Clinician Virtual-Care Experience Survey)	12–16 weeks
Ehrenreich-May J [26]	Unified Protocol for COVID-19 Parenting Stress (UP-COVID) intervention (cognitive behavioral therapy)	Sessions: 4 (à 30–90 min) Duration: NR	Group therapy	Delayed condition group (self-help guide + UP-COVID intervention after 6 weeks)	Anxiety (Overall Anxiety Severity and Impairment Scale) Depression (Overall Depression Severity and Impairment Scale) PTSD (Post-traumatic Stress Disorder Checklist for DSM-5)	6, 12 weeks
Lin X [27]	1. Cognitive and behavioral parent–child relationship intervention 2. Problem-solving and couple relationship improvement intervention	1. Sessions: 5 (à 2 h) 2. Sessions: 4 (à 2 h) Duration: NR	Online group therapy	/	Parent–child conflict (Conflict subscale of the Child–Parent Relationship Scale) Parent–child communication (Family Communication Scale) Parent–child relationship (Child–Parent Relationship Scale) Couple relationship quality (Quality of Marriage Index) Marital stability (Stability of Marriage Scale—Short Form)	2 weeks
Miklósi M [28]	Online parenting training programme 1. Training materials (text- or video-based) 2. Training materials + online forum 3. Training materials + online forum + therapist feedback	Sessions: 15–30-min daily activity Duration: 2 weeks	Online platform	/	Perceived stress (Perceived Stress Scale, four-item version) Psychological well-being (WHO Well-being Index) Parenting stress (Parental Stress Scale, shortened) Parental competence (Parental Sense of Competence Scale)	2 weeks, 1, 3 months
Francis L [29]	Tele-wellness supported digital toolkit (parenting support + self-care resources/digital learning games for children)	Sessions: daily/3 times per week (à 30 min) Duration: NR	Mobile application	/	Perceived level of stress (Perceived Stress Scale 10) Perceived level of informational support (Patient-Reported Outcomes Measurement Information System) Awareness of the Maryland early childhood family engagement framework and toolkit (Survey question) Social, emotional, and behavior functioning in children (Social Competence and Behavior Evaluation for Children—Short Form)	15 weeks

## Data Availability

All data generated or analyzed during this study are included in this published article.

## Abbreviations

CBT	Cognitive Behavioral Therapy
MERS	Middle East respiratory syndrome
PA	Physical activity
PTSD	Post-traumatic stress disorder
RCT	Randomized controlled trial
SARS	Severe acute respiratory syndrome
SD	Self-directed
SFBT	Solution Focused Brief Therapy
TARA	Training for Awareness Resilience and Action
UP-COVID	Unified Protocol for COVID-19 Parenting Stress

## Appendix A. MEDLINE Search Strategy

Database(s): Ovid MEDLINE(R) and Epub Ahead of Print, In-Process & Other Non-Indexed Citations, Daily and Versions(R) 1946-present

Results: 1319

**Table A1:** Search strategy

#	Searches
	exp coronavirus/
	exp coronavirus infections/
	covid *.mp.
	((corona * or corono *) adj1 (virus * or viral * or virinae *)).mp.
	(coronavirus * or coronovirus * or coronavirinae *).mp.
	(wuhan * or hubei * or huanan).mp.
	(2019-ncov or 2019ncov or ncov2019 or ncov-2019).mp.
	(covid-19 or covid19 or corvid-19 or corvid19).mp.
	(hcov-19 or hcov19).mp.
	(sars-cov-2 or sarscov-2 or sarscov2 or sars-cov2).mp.
	(sars-cov-1 or sarscov-1 or sarscov1 or sars-cov1).mp.
	(sarscov19 or sars-cov19 or sarscov-19 or sars-cov-19).mp.
	2019 novel *.mp.
	(ncov or n-cov).mp.
	cov.mp.
	(((respiratory * adj2 (symptom * or disease * or illness * or condition *)) or seafood market * or food market *) adj10 (wuhan * or hubei * or china * or chinese * or huanan *)).mp.
	((outbreak * or wildlife * or pandemic * or epidemic *) adj1 (china * or chinese * or huanan *)).mp.
	exp middle east respiratory syndrome coronavirus/
	middle east respiratory syndrome.mp.
	Mers *.mp.
	exp severe acute respiratory syndrome/
	severe acute respiratory syndrome.mp.
	sars *.mp.
	exp influenza a virus, h1n1 subtype/
	h1n1.mp.
	(h1n1 adj3 (virus or subtype)).mp.
	((influenza or influenza a or flu *) adj3 (pandem * or swine or pig * or h1n1 or 2009 or 2010)).mp.
	exp pandemics/
	Pandem *.mp.
	exp epidemics/
	epidemic *.mp.
32	or/1–31
	exp infant/
	infant *.mp.
	(baby or babies).mp.
	exp child/
	child *.mp.
	toddler *.mp.
	exp adolescent/
	adolescent *.mp.
	teen *.mp.
	minor *.mp.
	youth.mp.
	student *.mp.
	pupil *.mp.
	(paediatr * or pediatr *).mp.
	exp parents/
	parent *.mp.
	mother *.mp.
	matern *.mp.
	father *.mp.
	patern *.mp.
	exp family/
	famil *.mp.
	exp grandparents/
	(grandparent * or grand-parent *).mp.
	(grandmother * or grand-mother *).mp.
	(grandfather * or grand-father *).mp.
	exp parent-child relations/
	(parent * or mother * or father *) adj3 (child * or infant *) adj3 (relation * or bond *).mp.
	exp maternal behavior/
	exp paternal behavior/
	((parent * or matern * or patern *) adj3 behavio *).mp.
	exp caregivers/
	(caregiv * or care-giv *).mp.
	Carer *.mp.
	Educator *.mp.
68	or/33–67
	exp health education/
	(educat * adj3 (health or parent * or psych * or child * or service * or distan * or train * or program * or technolog * or physical * or continu * or support *)).mp.
	(learn * adj3 (support * or supervis * or continu * or distan * or material * or program * or resource * or assist *)).mp.
	exp health promotion/
	(promot * adj3 (mental health or skill *)).mp.
	exp teaching/
	exp teaching materials/
	(teach * adj3 (material * or aid * or method * or program * or session * or assist * or activit *)).mp.
	(assist * adj3 (child * or famil * or food * or nutrition *)).mp.
	(train * adj3 (physical * or intellect * or support * or program * or resource * or exercis *)).mp.
	exercise/
	(activ * adj3 (physical * or schedul *)).mp.
	self-help groups/
	(support * adj3 (group * or psych * or social * or pedagogical * or network *)).mp.
	(skill * adj3 (social * or emotional * or calm * or cop * or cognit * or digital *)).mp.
	exp behavior therapy/
	(behavio * adj3 (train * or treat * or therap * or manag *)).mp.
	(psych * adj3 (interven * or service *)).mp.
87	or/69–86
	exp mental health/
	(mental adj3 health).mp.
	((psych * or mental * or emotion * or social * or child *) adj3 (well-being or wellbeing)).mp.
	exp mental disorders/
	((psych * or mental * or depress *) adj3 (disease * or disorder * or ill * or disturb * or symptom *)).mp.
	exp stress, psychological/
	(stress * adj3 (psych * or mental * or emotion * or famil * or parent *)).mp.
	exp anxiety disorders/
	((stress * or anxiet *) adj3 disorder *).mp.
	(anxiet * or anxious).mp.
	exp depression/
	Depress *.mp.
	exp psychological trauma/
	((psych * or mental * or emotion *) adj3 (trauma * or damag * or injur * or harm *)).mp.
	exp stress disorders, post-traumatic/
	(posttraumatic or post-traumatic).mp.
	PTSD *.mp.
	exp psychological distress/
	((psych * or emotion *) adj3 (distress * or tension)).mp.
	exp sleep wake disorders/
	(sleep * adj3 (disorder * or disturb * or problem * or difficult * or pattern)).mp.
	exp child abuse/
	(abus * adj3 (physical * or emotional * or substance * or drug * or alcohol * or sexual * or child *)).mp.
	maltreat *.mp.
	mistreat *.mp.
	neglect *.mp.
	exp domestic violence/
	((domestic or famil * or partner or home) adj3 (viol * or abus * or conflict *)).mp.
	exp self concept/
	(self adj1 (concept or aware * or confront * or efficacy or image or perception or rat * or control * or esteem)).mp.
	exp child behavior/
	(behavio * adj3 (child * or infant * or disorder * or disturb * or problem *)).mp.
120	or/88–119
121	32 and 68 and 87 and 120
122	(english or german or russian or french).lg.
123	121 and 122

* Truncation.

## Appendix B. Embase Search Strategy

Database(s): Ovid Embase 1974-present

Results: 1964

**Table A2:** Search strategy.

#	Searches
	exp coronavirinae/
	exp coronavirus infection/
	Covid *.mp.
	((corona * or corono *) adj1 (virus * or viral * or virinae *)).mp.
	(coronavirus * or coronovirus * or coronavirinae *).mp.
	(wuhan * or hubei * or huanan).mp.
	(2019-ncov or 2019ncov or ncov2019 or ncov-2019).mp.
	(covid-19 or covid19 or corvid-19 or corvid19).mp.
	(hcov-19 or hcov19).mp.
	(sars-cov-2 or sarscov-2 or sarscov2 or sars-cov2).mp.
	(sars-cov-1 or sarscov-1 or sarscov1 or sars-cov1).mp.
	(sarscov19 or sars-cov19 or sarscov-19 or sars-cov-19).mp.
	2019 novel *.mp.
	(ncov or n-cov).mp.
	cov.mp.
	(((respiratory * adj2 (symptom * or disease * or illness * or condition *)) or seafood market * or food market *) adj10 (wuhan * or hubei * or china * or chinese * or huanan *)).mp.
	((outbreak * or wildlife * or pandemic * or epidemic *) adj1 (china * or chinese * or huanan *)).mp.
	exp middle east respiratory syndrome/
	middle east respiratory syndrome.mp.
	Mers *.mp.
	exp severe acute respiratory syndrome/
	severe acute respiratory syndrome.mp.
	Sars *.mp.
	exp influenza a virus (h1n1)/
	h1n1.mp.
	(h1n1 adj3 (virus or subtype)).mp.
	((influenza or influenza a or flu *) adj3 (pandem * or swine or pig * or h1n1 or 2009 or 2010)).mp.
	exp pandemic/
	Pandem *.mp.
	exp epidemic/
	epidemic *.mp.
32	or/1–31
	exp infant/
	Infant *.mp.
	(baby or babies).mp.
	exp child/
	child *.mp.
	toddler *.mp.
	exp adolescent/
	adolescent *.mp.
	teen *.mp.
	minor *.mp.
	youth.mp.
	student *.mp.
	pupil *.mp.
	(paediatr * or pediatr *).mp.
	exp parent/
	Parent *.mp.
	mother *.mp.
	matern *.mp.
	father *.mp.
	patern *.mp.
	exp family/
	famil *.mp.
	exp grandparent/
	(grandparent * or grand-parent *).mp.
	(grandmother * or grand-mother *).mp.
	(grandfather * or grand-father *).mp.
	exp child parent relation/
	(parent * or mother * or father *) adj3 (child * or infant *) adj3 (relation * or bond *).mp.
	exp parental behavior/
	((parent * or matern * or patern *) adj3 behavio *).mp.
	exp caregiver/
	(caregiv * or care-giv *).mp.
	Carer *.mp.
	educator*.mp.
67	or/33–66
	exp health education/
	exp parenting education/
	exp psychoeducation/
	(educat * adj3 (health or parent * or psych * or child * or service * or distan * or train * or program * or technolog * or physical * or continu * or support *)).mp.
	(learn * adj3 (support * or supervis * or continu * or distan * or material * or program * or resource * or assist *)).mp.
	exp health promotion/
	(promot * adj3 (mental health or skill *)).mp.
	exp teaching/
	(teach * adj3 (material * or aid * or method * or program * or session * or assist * or activit *)).mp.
	(assist * adj3 (child * or famil * or food * or nutrition *)).mp.
	exp training/
	(train * adj3 (physical * or intellect * or support * or program * or resource * or exercis *)).mp.
	(activ * adj3 (physical * or schedul *)).mp.
	exp support group/
	(support * adj3 (group * or psych * or social * or pedagogical * or network *)).mp.
	(skill * adj3 (social * or emotional * or calm * or cop * or cognit * or digital *)).mp.
	exp behaviour therapy/
	(behavio * adj3 (train * or treat * or therap * or manag *)).mp.
	(psych * adj3 (interven * or service *)).mp.
87	or/68–86
	exp mental health/
	(mental adj3 health).mp.
	exp psychological well-being/
	((psych * or mental * or emotion * or social * or child *) adj3 (well-being or wellbeing)).mp.
	exp mental disease/
	((psych * or mental * or depress *) adj3 (disease * or disorder* or ill * or disturb * or symptom*)).mp.
	exp mental stress/
	(stress * adj3 (psych * or mental * or emotion * or famil * or parent *)).mp.
	exp anxiety disorder/
	((stress * or anxiet *) adj3 disorder *).mp.
	(anxiet * or anxious).mp.
	exp depression/
	depress *.mp.
	exp psychotrauma/
	((psych * or mental * or emotion *) adj3 (trauma * or damag * or injur * or harm *)).mp.
	exp posttraumatic stress disorder/
	(posttraumatic or post-traumatic).mp.
	PTSD *.mp.
	exp distress syndrome/
	((psych * or emotion *) adj3 (distress * or tension)).mp.
	exp sleep disorder/
	(sleep * adj3 (disorder * or disturb * or problem * or difficult * or pattern)).mp.
	exp child abuse/
	(abus * adj3 (physical * or emotional * or substance * or drug * or alcohol * or sexual * or child *)).mp.
	maltreat *.mp.
	mistreat *.mp.
	neglect *.mp.
	exp domestic violence/
	((domestic or famil * or partner or home) adj3 (viol * or abus * or conflict *)).mp.
	exp self concept/
	(self adj1 (concept or aware * or confront * or efficacy or image or perception or rat * or control * or esteem)).mp.
	exp behavior disorder/
	(behavio * adj3 (disorder * or disturb * or problem *)).mp.
	exp child behavior/
	(behavio * adj3 (child * or infant* )).mp.
123	or/88–122
124	32 and 67 and 87 and 123
125	(english or german or russian or french).lg.
126	124 and 125

## Appendix C. PsycINFO Search Strategy

Database(s): EbscoHost PsycINFO 1887-present

Results: 1272

**Table A3:** Search strategy.

#	Searches
	TX coronavirus
	TX “coronavirus infection”
	TX covid *
	TX ((corona * or corono *) n1 (virus * or viral * or virinae *))
	TX (coronavirus * or coronovirus * or coronavirinae *)
	TX (wuhan * or hubei * or huanan)
	TX (2019-ncov or 2019ncov or ncov2019 or ncov-2019)
	TX (covid-19 or covid19 or corvid-19 or corvid19)
	TX (hcov-19 or hcov19)
	TX (sars-cov-2 or sarscov-2 or sarscov2 or sars-cov2)
	TX (sars-cov-1 or sarscov-1 or sarscov1 or sars-cov1)
	TX (sarscov19 or sars-cov19 or sarscov-19 or sars-cov-19)
	TX “2019 novel *”
	TX (ncov or n-cov)
	TX cov
	TX (((respiratory * n2 (symptom * or disease * or illness * or condition *)) or seafood market * or food market *) n10 (wuhan * or hubei * or china * or chinese * or huanan *))
	TX ((outbreak * or wildlife * or pandemic * or epidemic *) n1 (china * or chinese * or huanan *))
	TX “middle east respiratory syndrome”
	TX mers *
	TX “severe acute respiratory syndrome”
	TX sars *
	TX “influenza a virus (h1n1)”
	TX h1n1
	TX (h1n1 n3 (virus or subtype))
	TX ((influenza or influenza a or flu *) n3 (pandem * or swine or pig * or h1n1 or 2009 or 2010))
	TX pandem *
	TX epidemic*
28	or/1–27
	TX infant *
	TX (baby or babies)
	TX child *
	TX toddler *
	TX adolescent *
	TX teen *
	TX minor *
	TX youth
	TX student *
	TX pupil *
	TX (paediatr * or pediatr*)
	TX parent *
	TX mother *
	TX matern *
	TX father *
	TX patern *
	TX famil *
	TX (grandparent * or grand-parent *)
	TX (grandmother * or grand-mother *)
	TX (grandfather * or grand-father *)
	TX ((parent * or mother * or father *) n3 (child * or infant *) n3 (relation * or bond *))
	TX ((parent * or matern * or patern *) n3 behavio *))
	TX (caregiv * or care-giv *)
	TX carer *
	TX educator *
54	or/29–53
	TX (educat * n2 (health or parent * or psych * or child * or service * or distan * or train * or program * or technolog * or physical * or continu * or support *))
	TX (learn * n3 (support * or supervis * or continu * or distan * or material * or program * or resource * or assist *))
	TX “health promotion”
	TX (promot * n3 (mental health or skill *))
	TX teaching
	TX (teach * n3 (material * or aid * or method * or program * or session * or assist * or activit *))
	TX (assist * n3 (child * or famil * or food * or nutrition *))
	TX training
	TX (train * n3 (physical * or intellect * or support * or program * or resource * or exercis *))
	TX (activ * n3 (physical * or schedul *))
	TX (support * n3 (group * or psych * or social * or pedagogical * or network *))
	TX (skill * n3 (social * or emotional * or calm * or cop * or cognit * or digital *))
	TX (behavio * n3 (train * or treat * or therap * or manag *))
	TX (psych * n3 (interven * or service *))
69	or/55–68
	TX (mental n3 health)
	TX ((psych * or mental * or emotion * or social * or child *) n3 (well-being or wellbeing))
	TX ((psych * or mental * or depress *) n3 (disease * or disorder * or ill * or disturb * or symptom *))
	TX (stress * n3 (psych * or mental * or emotion * or famil * or parent *))
	TX ((stress * or anxiet *) n3 disorder *)
	TX (anxiet * or anxious)
	TX depress *
	TX ((psych * or mental * or emotion *) n3 (trauma * or damag * or injur * or harm *))
	TX (posttraumatic or post-traumatic)
	TX PTSD *
	TX ((psych * or emotion *) n3 (distress * or tension))
	TX (sleep * n3 (disorder * or disturb * or problem * or difficult * or pattern))
	TX (abus * n3 (physical * or emotional * or substance * or drug * or alcohol * or sexual * or child *))
	TX maltreat *
	TX mistreat *
	TX neglect *
	TX ((domestic or famil * or partner or home) n3 (viol * or abus * or conflict *))
	TX (self n1 (concept or aware * or confront * or efficacy or image or perception or rat * or control* or esteem))
	TX (behavio * n3 (disorder * or disturb * or problem *))
	TX (behavio * n3 (child * or infant *))
90	or/70–89
91	28 and 54 and 69 and 90
92	(english or german or russian or french).lg.
93	91 and 92

## Appendix D. CDC COVID-19 Research Articles Downloadable Database Search Strategy

Database(s): CDC COVID-19 Research Articles Downloadable Database (https://www.cdc.gov/library/researchguides/2019novelcoronavirus/researcharticles.html) 2020

Results: 2189

**Table A4:** Search strategy.

#	Searches
	infant
	baby
	babies
	child
	toddler
	adolescent
	teen
	minor
	youth
	student
	pupil
	paediatr
	pediatr
	parent
	mother
	matern
	father
	patern
	famil
	grandparent
	“grand-parent”
	grandmother
	“grand-mother”
	grandfather
	“grand-father”
	caregiv
	“care-giv”
	carer
	educator
30	or/1–29
	educat
	learn
	“health promotion”
	teach
	assist
	train
	“physical activity”
	“activity schedule”
	support
	skill
	behavio
	“psychological intervention”
	“psychological service”
44	or/31–43
	“mental health”
	“psychological well”
	“mental well”
	“emotional well”
	“social well”
	“child wel”
	“mental disease”
	“mental disorder”
	“mental illness”
	stress
	anxiety
	anxious
	depress
	trauma
	PTSD
	“psychological distress”
	“sleep disorder”
	abus
	maltreat
	mistreat
	neglect
	“domestic violence”
	“family violence”
	“family conflict”
	“partner violence”
	“self-concept”
	“self-awareness”
	“self-efficacy”
	“self-perception”
	“self-control”
	“self-esteem”
76	or/45–75
77	30 and 44 and 76

## Appendix E. WHO COVID-19 Database Search Strategy

Database(s): WHO COVID-19 Global literature on coronavirus disease (https://search.bvsalud.org/global-literature-on-novel-coronavirus-2019-ncov/) 2020

Results: 1011

**Table A5:** Search strategy.

#	Searches
	tw:(infant *)
	tw:((baby or babies))
	tw:(child *)
	tw:(toddler *)
	tw:(adolescent *)
	tw:(teen *)
	tw:(minor *)
	tw:(youth)
	tw:(student *)
	tw:(pupil *)
	tw:((paediatr * or pediatr *))
	tw:(parent *)
	tw:(mother *)
	tw:(matern *)
	tw:(father *)
	tw:(patern *)
	tw:(famil *)
	tw:((grandparent * or “grand-parent *”))
	tw:((grandmother * or “grand-mother *”))
	tw:((grandfather * or “grand-father *”))
	tw:((caregiv * or “care-giv *”))
	tw:(carer *)
	tw:(educator *)
24	or/1–23
	tw:(educat *)
	tw:(learn *)
	tw:(“health promotion”)
	tw:(teach *)
	tw:(assist *)
	tw:(train *)
	tw((“physical activity” or “activity schedule”))
	tw:(support *)
	tw:(skill *)
	tw:(behavio *)
	tw:((“psychological intervention *” or “psychological service *”))
36	or/25–35
	tw:(“mental health”)
	tw:((“psychological well *” or “mental well *” or “emotional well *” or “social well *” or “child wel *”))
	tw:((“mental disease” or “mental disorder” or “mental illness”))
	tw:(stress *)
	tw:((anxiet * or anxious))
	tw:(depress *)
	tw:(trauma *)
	tw:(PTSD *)
	tw:(“psychological distress *”)
	tw:(“sleep disorder”)
	tw:(abus *)
	tw:(maltreat *)
	tw:(mistreat *)
	tw:(neglect *)
	tw:((“domestic violence” or “family violence” or “family conflict” or “partner violence”))
	tw:((“self-concept” or “self-awareness” or “self-efficacy” or “self-perception” or “self-control*” or “self-esteem*”))
53	or/7–52
54	24 and 36 and 53

## Appendix F. Cochrane COVID-19 Study Register Search Strategy

Database(s): Cochrane COVID-19 Study Register (https://covid-19.cochrane.org/) 2020

Results: 434

Default search setting(s): interventional studies (clinical trials)

**Table A6:** Search strategy.

#	Searches
	Infant *
	baby or babies
	child *
	toddle *
	adolescent *
	teen *
	minor *
	youth
	student *
	pupil *
	paediatr * or pediatr *
	parent *
	mother *
	matern *
	father *
	patern *
	famil *
	grandparent * or “grand-parent *”
	grandmother * or “grand-mother *”
	grandfather * or “grand-father *”
	caregiv * or “care-giv *”
	carer *
	educator *
24	or/1–23
	educat *
	learn *
	“health promotion”
	teach *
	assist *
	train *
	tw“physical activity” or “activity schedule”
	support *
	skill *
	behavio *
	“psychological intervention *” or “psychological service *”
36	or/5–35
	“mental health”
	“psychological well *” or “mental well *” or “emotional well *” or “social well *” or “child wel *”
	“mental disease” or “mental disorder” or “mental illness”
	stress *
	anxiet * or anxious
	depress *
	trauma *
	PTSD *
	“psychological distress *”
	“sleep disorder”
	abus *
	maltreat *
	mistreat *
	neglect *
	“domestic violence” or “family violence” or “family conflict” or “partner violence”
	“self-concept” or “self-awareness” or “self-efficacy” or “self-perception” or “self-control *” or “self-esteem *”
53	or/37–52
54	24 and 36 and 53

## Appendix G. ClinicalTrials.gov Search Strategy

Database(s): ClinicalTrials.gov, National Institutes of Health (NIH) (https://clinicaltrials.gov) 2020

Results: 46

Default search setting(s): covid-19, child (birth-17), interventional studies (clinical trials)

**Table A7:** Search strategy.

#	Searches
	psychological
	mental
	emotional
	social
	stress
	anxiety
	depression
	trauma
9	**or/1–8**

## Appendix H. EU Clinical Trials Register Search Strategy

Database(s): EU Clinical Trials Register (https://www.clinicaltrialsregister.eu) 2020

Results: 19

Default search setting(s): Adolescent, children, infant and toddler, newborn, under 18 (age range)

**Table A8:** Search strategy.

#	Searches
	adolescent
	children
	infant and toddler
	newborn
	under 18
6	or/1–5
	covid-19
8	6 and 7

## Appendix I. German Clinical Trials Register (DRKS) Search Strategy

Database(s): German Clinical Trials Register (DRKS) (https://www.drks.de/drks_web/) 2020

Results: 55

Default search setting(s): interventional studies (clinical trials)

**Table A9:** Search strategy.

#	Searches
	0–6 years
	7–14 years
	15–17 years
4	or/1–3
	covid–19
6	4 and 5

## Appendix J. Data Extraction Form

We used an electronic data extraction form in Microsoft Excel, which included the following items:

Study information:

- Author(s)
- Study title
- Publication year
- Study source (journal, report, pre-print publication)

Study design:

- Study type (e.g., cross-sectional study, mixed methods)
- Objectives/purpose

Population/context:

- Population studied (characteristics, total number of participants)
- Region/country affected by disease outbreak
- Disease outbreak (e.g., COVID-19, H1N1 influenza)
- Study setting
- Pandemic control measure(s) in place (e.g., quarantine, school closure)

Intervention:

- Broad measure category (e.g., training, support group)
- Description of specific measure(s) (e.g., smartphone-based psychoeducation)
- Mode of delivery (e.g., online, face to face)
- Implementation of measure(s)
- Comparison group
- Comments

Outcomes:

- Outcome category (e.g., mental health)
- Description of outcome (e.g., depression)
- Link between intervention and outcome
- Follow-up
- Effect measure
- Total number of participants
- Outcome in the intervention group
- Participants in the intervention group
- Outcome in the comparison group
- Participants in the comparison group
- Comments
